# Dietary n-3:n-6 fatty acid ratios differentially influence hormonal signature in a rodent model of metabolic syndrome relative to healthy controls

**DOI:** 10.1186/1743-7075-7-53

**Published:** 2010-06-28

**Authors:** Paul R Burghardt, Elyse S Kemmerer, Bradley J Buck, Andrew J Osetek, Charles Yan, Lauren G Koch, Steven L Britton, Simon J Evans

**Affiliations:** 1 Molecular and Behavioral Neuroscience Institute, The University of Michigan, MBNI 2028, 205 Zina Pitcher Place, Ann Arbor, MI 48109, USA; 2 Department of Psychiatry, The University of Michigan, BSRB 5059,109 Zina Pitcher Place, Ann Arbor, MI 48109, USA; 3 Department of Anesthesiology, The University of Michigan, BSRB 2021, 109 Zina Pitcher Place, Ann Arbor, MI, USA

## Abstract

Dietary ratios of omega-3 (n-3) to omega-6 (n-6) polyunsaturated fatty acids (PUFAs) have been implicated in controlling markers of the metabolic syndrome, including insulin sensitivity, inflammation, lipid profiles and adiposity. However, the role of dietary PUFAs in regulating energy systems in healthy relative to metabolic diseased backgrounds has not been systematically addressed. We used dietary manipulation of n-3 to n-6 PUFA ratios in an animal model of metabolic syndrome and a related healthy line to assay feeding behavior and endocrine markers of feeding drive and energy regulation. Two related lines of rodents with a healthy and a metabolic syndrome phenotype were fed one of two isocaloric diets, comprised of either a 1:1 or a 1:30 n-3 to n-6 ratio, for 30 days. Food intake and weight gain were monitored; and leptin, ghrelin, adiponectin and a suite of hypothalamic neuropeptides involved in energy regulation were assayed following the dietary manipulation period. There was no difference in caloric intake or weight gain between diet groups, however there was a significant interaction between diet and phenotypic line on central and peripheral markers of energy homeostasis. Thus serum levels of leptin, acylated-ghrelin and adiponectin, and mRNA levels of the anorexigenic hypothalamic neuropeptide, cocaine-amphetamine related transcript (CART), showed differential, dietary responses with HCR rats showing an increase in anorexigenic signals in response to unbalanced n-3:6 ratios, while LCR did not. These data are the first to demonstrate that a rodent line with a metabolic syndrome-like phenotype responds differentially to dietary manipulation of n-3 and n-6 fatty acids relative to a related healthy line with regard to endocrine markers of energy homeostasis. The dietary n-3:n-6 ratios used in this experiment represent extreme points of natural human diets, however the data suggest that optimal recommendations regarding omega-3 and omega-6 intake may have differing effects in healthy subjects relative to metabolic syndrome patients. Further research is necessary to establish these responses in human populations.

## Findings

A dietary constituent that may have remediating effects on metabolic syndrome are the polyunsaturated fatty acids (PUFA) in the omega-3 (n-3) class. Supplementation with the long chain n-3 PUFA has been shown to decrease insulin resistance, triglyceride levels, heart rate, and blood pressure, and increase HDL cholesterol levels [1]. Conversely, omega-6 (n-6) PUFA, which compete with n-3s for several physiological processes and are abundant in the western diet [2], can increase inflammatory signals and have been associated with cardiovascular heart disease (CHD) [3].

Given the many opposing effects of n-3s compared to n-6s, recent studies have begun to dissect how the dietary ratios of these PUFA influence health and disease. While many n-6 derived eicosanoids propagate inflammatory signals, many n-3 derived eicosanoids are less inflammatory and even anti-inflammatory by competition [4]. Therefore, diets with higher n-6 to n-3 ratios may contribute to the pathology of metabolic syndrome through inflammatory processes and other currently unrecognized mechanisms. It should be pointed out that the relationship between n-3 and n-6 PUFA is complex and they are not always in opposition. For example, the n-6 derived lipoxins have anti-inflammatory effects. Nevertheless, the dietary-influenced tissue ratios of n-3 to n-6 PUFA are important in their contribution to health and disease [3].

Recently, a naturalistic rodent model of metabolic syndrome was developed via selective breeding for intrinsic running capacity [5]. This selection strategy resulted in animals with high or low intrinsic running capacity, referred to as high capacity runners (HCR) and low capacity runners (LCR), respectively. After several generations of selection and breeding, LCR rats developed numerous markers of metabolic syndrome, including, elevated LDL cholesterol, blood pressure, triglycerides, fasting glucose, insulin, C-reactive protein, and visceral adiposity. Conversely, HCR animals appeared physiologically healthy with a number of their metabolic parameters and intrinsic treadmill capacity falling within ranges reported for standard inbred strains [6,7].

In the present study, we fed HCR and LCR lines one of two isocaloric diets, identical in total macronutrient composition. They differed only in the fat source to establish an unbalanced (30:1 n-6 to n-3) or balanced (1:1 n-6 to n-3) ratio of n-6s to n-3s, to mirror the extremes of the range of ratios reported in different human populations [3,8]. Following dietary manipulation, we evaluated food intake and weight gain, circulating hormone levels related to energy management, and hypothalamic gene expression of neuropeptides involved in feeding and energy expenditure.

### Animals and diets

HCR and LCR rats were selectively bred for intrinsic running capacity as previously described [5]. Generation 19 adult male HCR (n = 16) and LCR (n = 16) rats were housed in pairs and randomly assigned to receive one of two diets for 30 days. Balanced (1:1) and unbalanced (30:1) n-6:n-3 fatty acid diets were based on Harlan Teklad's (Madison, Wisconsin) purified diet and customized to contain the specified PUFA ratios (catalog #'s TD 06212 (1:1 diet) and TD 06213 (30:1 diet)), as outlined in Table 1. The diets were calorically identical with equal amounts of total protein, fat and carbohydrate. Rats were weighed and their average food consumption (per cage) was measured every 3 days. Rats were euthanized by decapitation, their brains were removed, and trunk blood collected as approved by The University Committee on Use and Care of Laboratory Animals at the University of Michigan.

**Table 1:** Diet Composition

	Unbalanced Diet		Balanced Diet	
**Ingedient**	**g/kg**	****%****	**g/kg**	****%****

Casein	200.0	20	200.0	20

DL-Methionine	3.0	0.3	3.0	0.3

Corn Starch	388.4	38.8	388.5	38.8488

Maltodextrin (Lo-Dex)	100.0	10	100.0	10

Sucrose	150.0	15	150.0	15

Fish Oil	4.3	0.43	43.7	4.37

Safflower Oil	55.7	5.57	16.3	1.63

Cellulose (Fiber)	47.0	4.7	47.0	4.7

Mineral Mix, AIN-93G-MX	35.0	3.5	35.0	3.5

Calcium Phosphate Dibasic	4.0	0.4	4.0	0.4

Vitamin Mix, AIN-93-VX	10.0	1	10.0	1

Choline Bitartrate	2.5	0.25	2.5	0.25

TBHQ (Antioxidant)	0.012	0.0012	0.012	0.0012

Totals (g/kg)	1000.0	100.0	1000.0	100.00

Diet %				

kcal/kg	3731.21		3731.21	

kcal/g	3.73		3.73	

kcal %				

Ca:P	1.61		1.61	

				

Fat Composition (g/kg)	Unbalanced Diet		Balanced Diet	

SFA	6.79		15.85	

MUFA	9.59		14.46	

PUFA	44.78		27.66	

18:2	42.97		13.37	

18:3	0.10		0.81	

20:5	0.53		5.33	

22:6	0.47		4.73	

n-6	43.07		14.37	

n-3	1.43		14.26	

### Blood plasma feeding hormone detection

Blood plasma was isolated by centrifugation at 3000 rpm for 10 min and stored at -80°C. Commercially-available enzyme immunoassay kits were used to quantify the plasma levels of: leptin (Assay Designs, Ann Arbor, MI), acylated-ghrelin (Cayman Chemical, Ann Arbor, MI) and adiponectin (Alpco Diagnostics, Salem, NH), according to the manufacturer's instructions.

### In situ hybridization analysis

Brains were sectioned and prepared for in situ hybridization and processed as previously described [9]. Probes included ^35^S-labeled-antisense probes for the following transcripts: neuropeptide-Y (NPY; accession number: M20373), preproorexin (accession number: NM013179), agouti-related peptide (AgRP; accession number: AF206017), cocaine-amphetamine regulated transcript (CART; accession number: NM017110), and proopiomelanocortin (POMC; accession number: J00759).

### Statistical analysis

The dependent variables of body weight, food intake, hormone level (leptin, ghrelin, adiponectin), and mRNA expression (AgRP, CART, MCH, NPY, orexin and POMC) were all analyzed by two-way analysis of variance (ANOVA). The independent variables of diet (balanced or unbalanced) and line (HCR or LCR) consisted of two levels. Tukey's honestly significant difference (HSD) was used for post hoc analysis to determine differences among individual groups, but only when significant interactions were found. For all analyses, Statistical Analysis Software (SAS) package (Cary, NC) was used, and statistical significance was set at p < 0.05.

### Food intake and weight gain are similarly affected by balanced and unbalanced n-3:n-6 ratios

**Figure 1:**
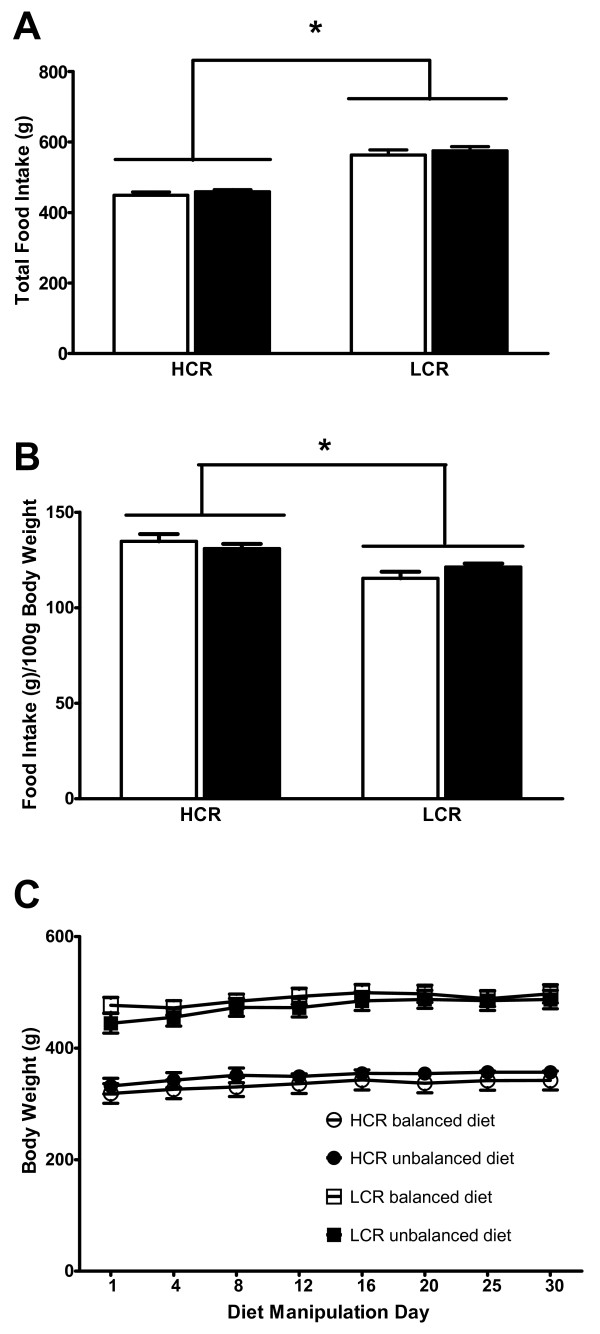
**Food Intake and Weight Gain**. A) Total food intake of HCR and LCR lines for each diet during the 30 day period. LCRs ate significantly more than HCRs but within a line, both diets were consumed in equal amounts. B) Total food intake adjusted per gram body weight of HCRs was significantly greater than that of LCRs. C) Weight gain over time did not differ between two diets within a line. LCRs weighed significantly more than HCRs at all points.

Across both diets, LCR rats consumed more food than HCR rats as indicated by a main effect of line (p < 0.0001; Figure 1a). When feeding was calculated as a function of body weight, HCR rats on both diets ate more food per body weight than LCR rats (p = 0.0003; Figure 1b). There was no difference in food intake across diet groups within each line. Therefore, overall calorie and macronutrient intake were the same across diets within a line, making the results given below directly comparable across diets. There were no significant effects of line or diet on weight gain (Figure 1c).

### Circulating hormone levels respond differently to dietary manipulation

**Figure 2:**
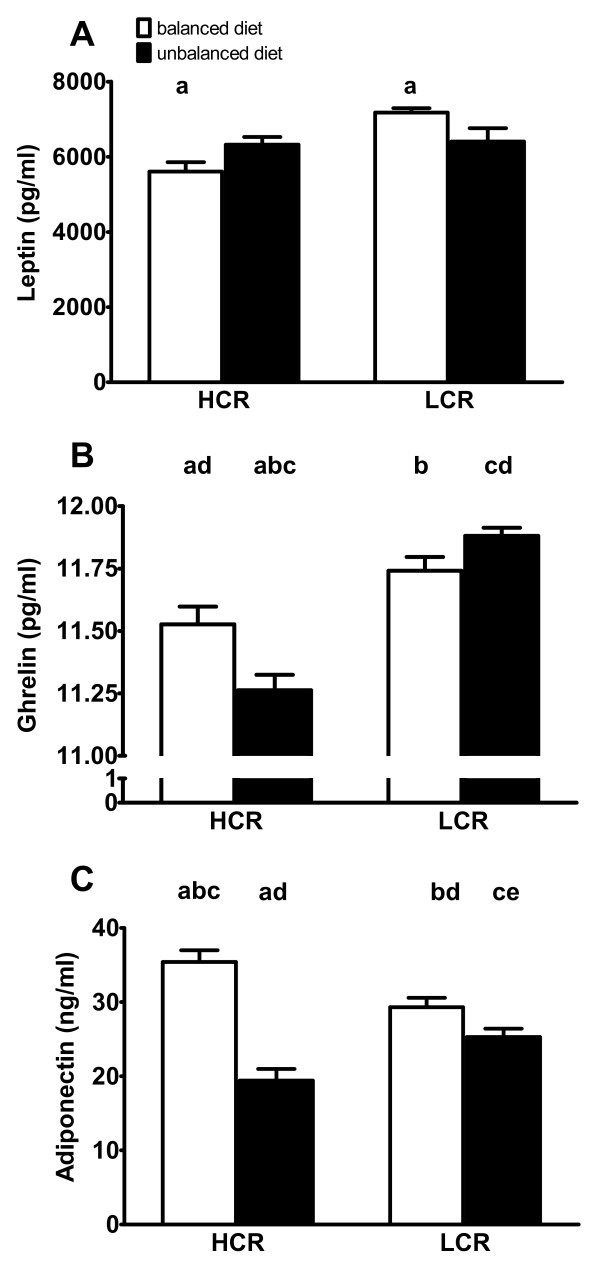
**Serum Levels of Energy Regulating Hormones**. Bars with the same identifying letters above them were significantly different from each other at the p < 0.05 level. A) There was a significant line by diet interaction for leptin levels assayed at the end of the dietary period. Post-hoc analysis revealed that levels of HCRs on the balanced diet had lower levels of leptin than LCRs on the same diet. B) There was a significant line by diet interaction for acylated-ghrelin levels assayed at the end of the dietary period. Post-hoc analysis revealed that acylated-ghrelin levels in HCRs on the balanced diet were significantly greater than HCRs on the unbalanced diet and significantly lower than LCRs on the unbalanced diet. Furthermore, acylated-ghrelin levels in HCRs on the unbalanced diet were significantly lower than in all other groups. C) There was a significant line by diet interaction for adiponectin levels assayed at the end of the dietary period. Post-hoc analysis revealed that adiponectin serum levels in HCRs on the balanced diet were significantly greater than all other groups and the HCRs on the unbalanced diet were significantly lower than all other groups.

There was a significant interaction between rodent line and diet on plasma leptin levels (p = 0.0059; Figure 2a). On the balanced diet, the HCR rats had significantly lower levels of leptin than the LCR rats (p < 0.05), however, there was no difference between leptin levels of HCR and LCR rats consuming the unbalanced diet. Similarly, a significant interaction between line and diet was also detected for acylated-ghrelin levels (p = 0.0014; Figure 2b). Post-hoc tests revealed that HCR rats on the balanced diet had higher levels of acylated-ghrelin than HCR rats on the unbalanced diet (p < 0.05), but there was no significant difference between LCR rats across the two diets. In addition, there was a main effect of line, where HCR rats had lower levels of acylated-ghrelin than LCR rats (P < 0.05). Finally, a significant interaction was found between line and diet on adiponectin levels (p = 0.0003; Figure 2c). Post hoc analysis revealed that HCR rats on the balanced diet had higher levels of adiponectin than any other group, while HCR rats on the unbalanced diet had adiponectin levels lower than any other group (p < 0.05).

### Dietary manipulation differentially affects CART expression in LCR and HCR rats

**Figure 3:**
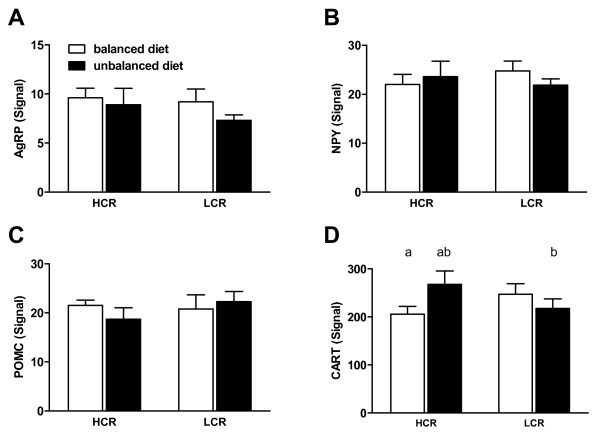
**Messenger RNA Expression of Energy Regulating Neuropeptides in the Hypothalamus**. Bars with the same identifying letters above them were significantly different from each other at the p < 0.05 level. A - C) AgRP, NPY and POMC mRNA levels, respectively, in the arcuate nucleus of the hypothalamus following the dietary manipulation period of 30 days. No significant differences were found across groups. D) CART mRNA expression in the arcuate nucleus of the hypothalamus following the 30 day dietary manipulation. There was a significant line by diet interaction for CART mRNA expression. Post-hoc analysis showed that CART mRNA in HCRs on the balanced diet was expressed at significantly lower levels than in HCRs on the unbalanced diet. Furthermore, CART mRNA expression in HCRs on the unbalanced diet was significantly higher than CART mRNA levels in LCRs on the unbalanced diet.

A significant interaction was detected between diet and line on transcript levels of CART in the arcuate nucleus of the hypothalamus (p = 0.0423; Figure 3d). Post-hoc testing revealed that CART expression was higher in HCR rats on the unbalanced diet compared to either the HCR rats on the balanced diet or the LCR rats on the unbalanced diet. No significant changes were detected in hypothalamic expression of POMC, AgRP, NPY or orexin.

The results of this experiment are the first, to our knowledge, to show that manipulation of dietary n-6:n-3 PUFA ratios differentially affects an animal model of metabolic syndrome (LCR) as compared with a healthy related strain of rats (HCR). Peripheral and central drivers of energy homeostasis were differentially altered in HCR and LCR rats in response to unbalanced n-6:n-3 dietary ratios. Specifically, serum leptin, acylated-ghrelin and adiponectin were responsive to dietary manipulation in a line-specific manner. In the hypothalamus expression of the anorexigenic neuropeptide, CART, was different in the HCR and LCR animals following dietary n-6:n-3 manipulation. These data are indicative of an overall increase in orexigenic signal in a rat model of MetS (LCR) fed unbalanced relative to a balanced dietary n-6:n-3 ratio; and conversely, a significant decrease in orexigenic signal in healthy rodents (HCR) fed and unbalanced relative to a balanced dietary n-6:n-3 ratio.

Paradoxically, we did not see these hormonal signatures translate into increased feeding behavior. However, this might be explained in two ways. First, these measurements were all terminal endpoints and it's possible that the LCR rats on the unbalanced diet would have begun to eat more in time. Second, higher-level central control of feeding behavior [10,11] may have superseded these changes to inhibit increased feeding behavior in the LCR animals on the unbalanced diet. In fact, we found no changes in orexin, NPY or POMC in hypothalamic feeding circuits, supporting this hypothesis.

In summary, the main finding in this study is the opposing response on several measures of energy management between a rodent model of MetS and a related healthy line, to dietary manipulation of n-6:n-3 fatty acid ratios. Taken as a whole, this study indicates that unbalanced dietary ratios of n-6:n-3 may exacerbate physiological conditions that contribute to disease in an at risk model of MetS, whereas a related healthy rodent line showed potential compensatory responses to counteract consumption of unbalanced dietary n-6:n-3 levels. Furthermore, these data suggest that dietary advice given to healthy individuals, regarding PUFA intake, may differentially affect those with metabolic syndrome.

## Competing interests

The authors declare that they have no competing interests.

## Statement of Author Contributions are as Follows

P.R.B., E.S.K and S.J.E designed research; P.R.B, E.S.K, B.J.B, A.J.O, C.Y. and S.J.E conducted research; S.L.B and L.G.K. provided essential materials; P.R.B, E.S.K., and S.J.E. analyzed data; P.R.B., E.S.K. and S.J.E wrote paper, P.R.B and S.J.E. had primary responsibility for final content. All authors read and approved the final manuscript.
